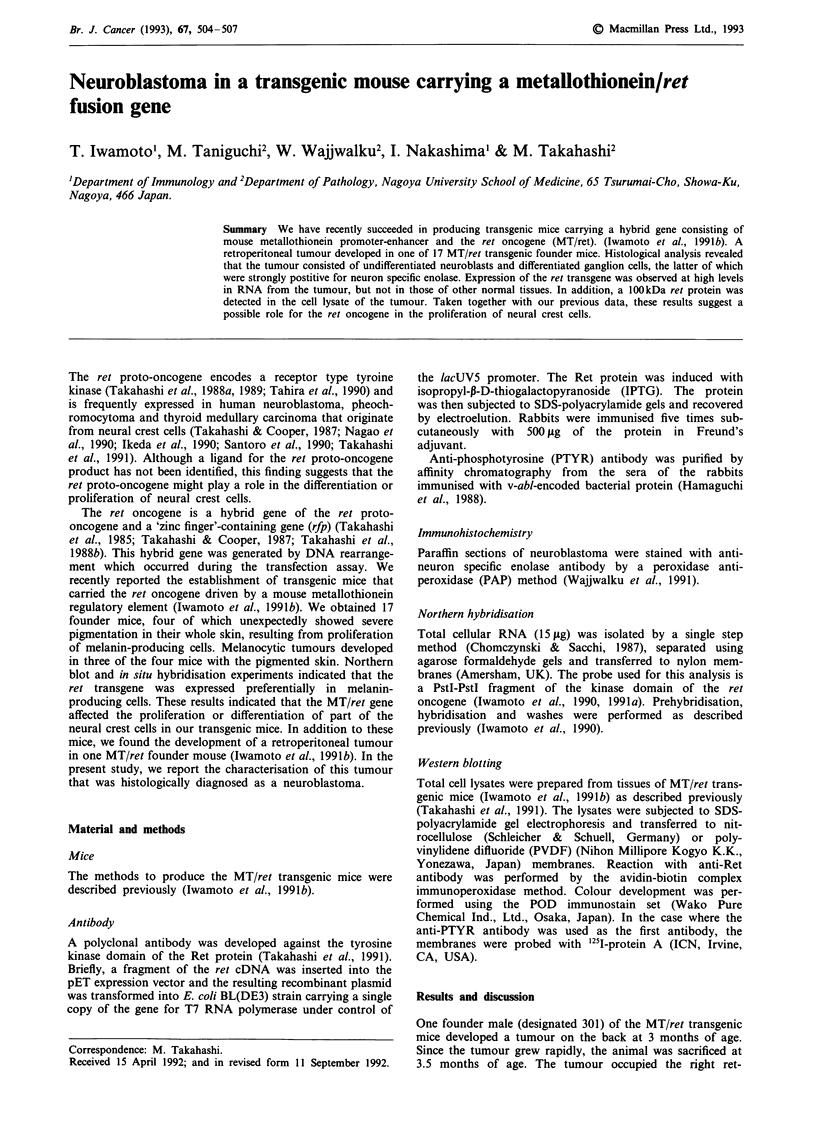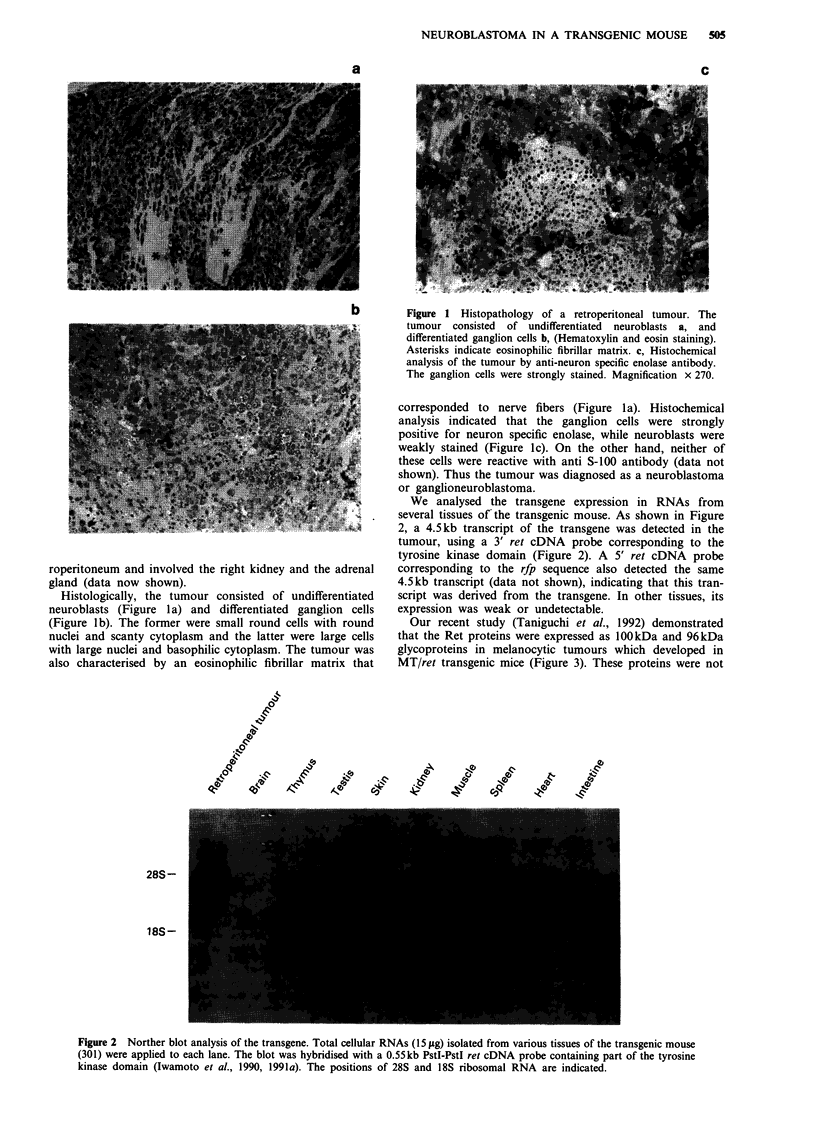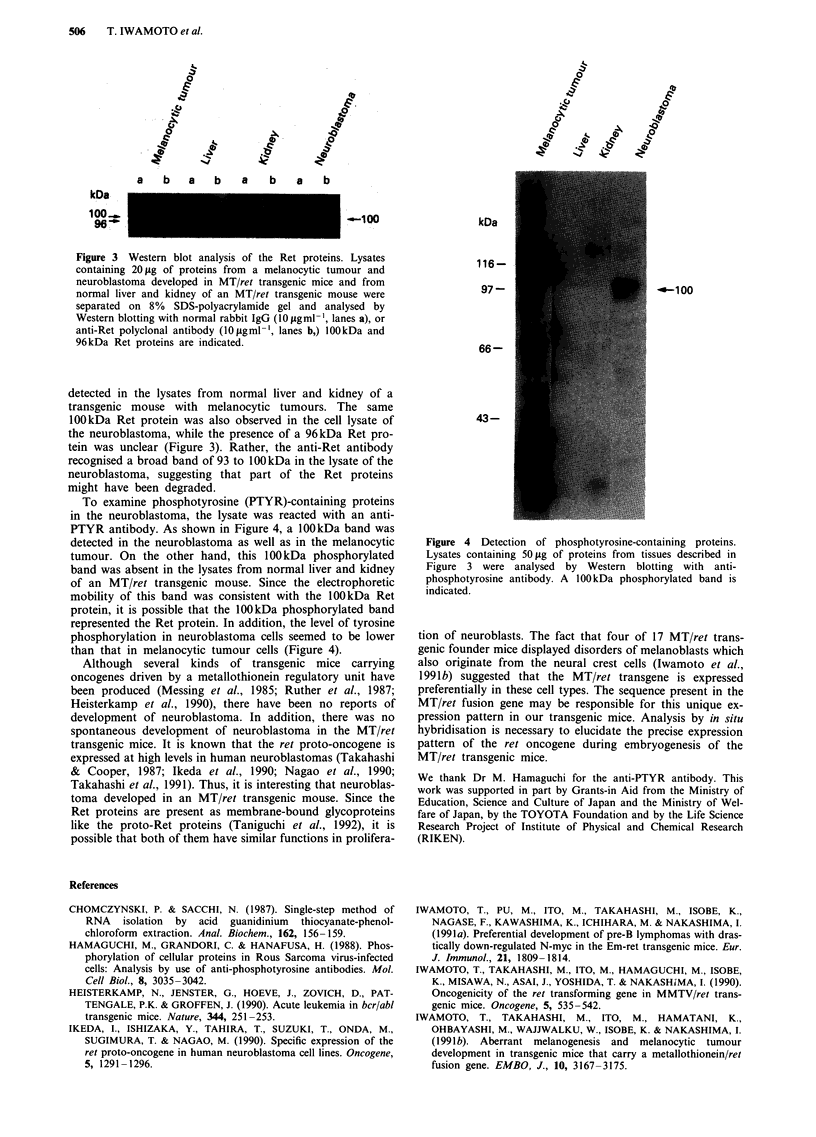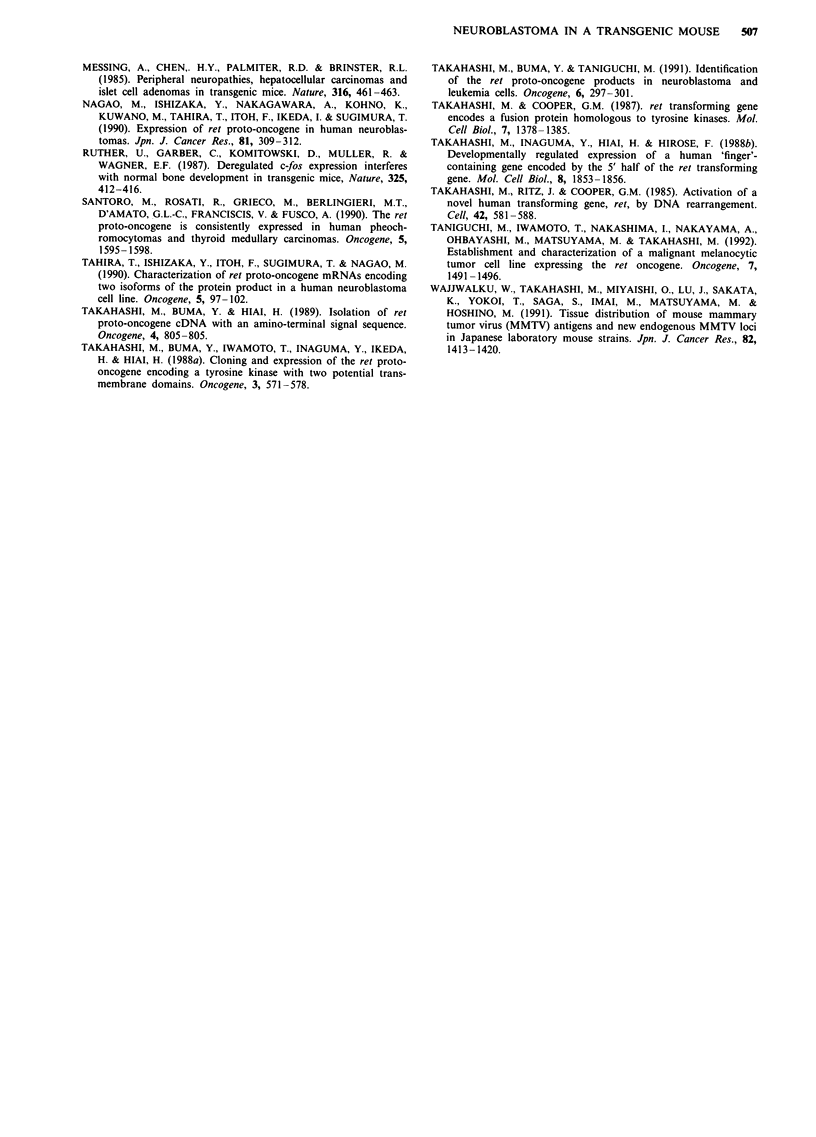# Neuroblastoma in a transgenic mouse carrying a metallothionein/ret fusion gene.

**DOI:** 10.1038/bjc.1993.94

**Published:** 1993-03

**Authors:** T. Iwamoto, M. Taniguchi, W. Wajjwalku, I. Nakashima, M. Takahashi

**Affiliations:** Department of Immunology, Nagoya University School of Medicine, Japan.

## Abstract

**Images:**


Br. J. Cancer (1993), 67, 504–507 © Macmillan Press Ltd., 1993

### Neuroblastoma in a transgenic mouse carrying a metallothionein/*ret* fusion gene

T. Iwamoto[1], M. Taniguchi[2], W. Wajjwalku[2], I. Nakashima[1] & M. Takahashi[2]

[1]*Department of Immunology and* [2]*Department of Pathology, Nagoya University School of Medicine, 65 Tsurumai-Cho, Showa-Ku, Nagoya, 466 Japan.*

**Summary** We have recently succeeded in producing transgenic mice carrying a hybrid gene consisting of mouse metallothionein promoter-enhancer and the *ret* oncogene (MT/ret). (Iwamoto *et al.*, 1991*b*). A retroperitoneal tumour developed in one of 17 MT/*ret* transgenic founder mice. Histological analysis revealed that the tumour consisted of undifferentiated neuroblasts and differentiated ganglion cells, the latter of which were strongly postitive for neuron specific enolase. Expression of the *ret* transgene was observed at high levels in RNA from the tumour, but not in those of other normal tissues. In addition, a 100 kDa *ret* protein was detected in the cell lysate of the tumour. Taken together with our previous data, these results suggest a possible role for the *ret* oncogene in the proliferation of neural crest cells.

The *ret* proto-oncogene encodes a receptor type tyroine kinase (Takahashi *et al.*, 1988*a*, 1989; Tahira *et al.*, 1990) and is frequently expressed in human neuroblastoma, pheochromocytoma and thyroid medullary carcinoma that originate from neural crest cells (Takahashi & Cooper, 1987; Nagao *et al.*, 1990; Ikeda *et al.*, 1990; Santoro *et al.*, 1990; Takahashi *et al.*, 1991). Although a ligand for the *ret* proto-oncogene product has not been identified, this finding suggests that the *ret* proto-oncogene might play a role in the differentiation or proliferation of neural crest cells.

The *ret* oncogene is a hybrid gene of the *ret* proto-oncogene and a 'zinc finger'-containing gene (*rfp*) (Takahashi *et al.*, 1985; Takahashi & Cooper, 1987; Takahashi *et al.*, 1988*b*). This hybrid gene was generated by DNA rearrangement which occurred during the transfection assay. We recently reported the establishment of transgenic mice that carried the *ret* oncogene driven by a mouse metallothionein regulatory element (Iwamoto *et al.*, 1991*b*). We obtained 17 founder mice, four of which unexpectedly showed severe pigmentation in their whole skin, resulting from proliferation of melanin-producing cells. Melanocytic tumours developed in three of the four mice with the pigmented skin. Northern blot and *in situ* hybridisation experiments indicated that the *ret* transgene was expressed preferentially in melanin-producing cells. These results indicated that the MT/*ret* gene affected the proliferation or differentiation of part of the neural crest cells in our transgenic mice. In addition to these mice, we found the development of a retroperitoneal tumour in one MT/*ret* founder mouse (Iwamoto *et al.*, 1991*b*). In the present study, we report the characterisation of this tumour that was histologically diagnosed as a neuroblastoma.

### Material and methods

### *Mice*

The methods to produce the MT/*ret* transgenic mice were described previously (Iwamoto *et al.*, 1991*b*).

### *Antibody*

A polyclonal antibody was developed against the tyrosine kinase domain of the Ret protein (Takahashi *et al.*, 1991). Briefly, a fragment of the *ret* cDNA was inserted into the pET expression vector and the resulting recombinant plasmid was transformed into *E. coli* BL(DE3) strain carrying a single copy of the gene for T7 RNA polymerase under control of the *lac*UV5 promoter. The Ret protein was induced with isopropyl-β-D-thiogalactopyranoside (IPTG). The protein was then subjected to SDS-polyacrylamide gels and recovered by electroelution. Rabbits were immunised five times subcutaneously with 500 µg of the protein in Freund's adjuvant.

Anti-phosphotyrosine (PTYR) antibody was purified by affinity chromatography from the sera of the rabbits immunised with v-*abl*-encoded bacterial protein (Hamaguchi *et al.*, 1988).

### *Immunohistochemistry*

Paraffin sections of neuroblastoma were stained with anti-neuron specific enolase antibody by a peroxidase anti-peroxidase (PAP) method (Wajjwalku *et al.*, 1991).

### *Northern hybridisation*

Total cellular RNA (15 µg) was isolated by a single step method (Chomczynski & Sacchi, 1987), separated using agarose formaldehyde gels and transferred to nylon membranes (Amersham, UK). The probe used for this analysis is a PstI-PstI fragment of the kinase domain of the *ret* oncogene (Iwamoto *et al.*, 1990, 1991*a*). Prehybridisation, hybridisation and washes were performed as described previously (Iwamoto *et al.*, 1990).

### *Western blotting*

Total cell lysates were prepared from tissues of MT/*ret* transgenic mice (Iwamoto *et al.*, 1991*b*) as described previously (Takahashi *et al.*, 1991). The lysates were subjected to SDS-polyacrylamide gel electrophoresis and transferred to nitrocellulose (Schleicher & Schuell, Germany) or polyvinylidene difluoride (PVDF) (Nihon Millipore Kogyo K.K., Yonezawa, Japan) membranes. Reaction with anti-Ret antibody was performed by the avidin-biotin complex immunoperoxidase method. Colour development was performed using the POD immunostain set (Wako Pure Chemical Ind., Ltd., Osaka, Japan). In the case where the anti-PTYR antibody was used as the first antibody, the membranes were probed with $^{125}$I-protein A (ICN, Irvine, CA, USA).

### Results and discussion

One founder male (designated 301) of the MT/*ret* transgenic mice developed a tumour on the back at 3 months of age. Since the tumour grew rapidly, the animal was sacrificed at 3.5 months of age. The tumour occupied the right ret-

Correspondence: M. Takahashi.
Received 15 April 1992; and in revised form 11 September 1992.

**Figure 1** Histopathology of a retroperitoneal tumour. The tumour consisted of undifferentiated neuroblasts **a**, and differentiated ganglion cells **b**, (Hematoxylin and eosin staining). Asterisks indicate eosinophilic fibrillar matrix. **c**, Histochemical analysis of the tumour by anti-neuron specific enolase antibody. The ganglion cells were strongly stained. Magnification × 270.

roperitoneum and involved the right kidney and the adrenal gland (data now shown).

Histologically, the tumour consisted of undifferentiated neuroblasts (Figure 1a) and differentiated ganglion cells (Figure 1b). The former were small round cells with round nuclei and scanty cytoplasm and the latter were large cells with large nuclei and basophilic cytoplasm. The tumour was also characterised by an eosinophilic fibrillar matrix that corresponded to nerve fibers (Figure 1a). Histochemical analysis indicated that the ganglion cells were strongly positive for neuron specific enolase, while neuroblasts were weakly stained (Figure 1c). On the other hand, neither of these cells were reactive with anti S-100 antibody (data not shown). Thus the tumour was diagnosed as a neuroblastoma or ganglioneuroblastoma.

We analysed the transgene expression in RNAs from several tissues of the transgenic mouse. As shown in Figure 2, a 4.5 kb transcript of the transgene was detected in the tumour, using a 3′ *ret* cDNA probe corresponding to the tyrosine kinase domain (Figure 2). A 5′ *ret* cDNA probe corresponding to the *rfp* sequence also detected the same 4.5 kb transcript (data not shown), indicating that this transcript was derived from the transgene. In other tissues, its expression was weak or undetectable.

Our recent study (Taniguchi *et al.*, 1992) demonstrated that the Ret proteins were expressed as 100 kDa and 96 kDa glycoproteins in melanocytic tumours which developed in MT/*ret* transgenic mice (Figure 3). These proteins were not

**Figure 2** Norther blot analysis of the transgene. Total cellular RNAs (15 μg) isolated from various tissues of the transgenic mouse (301) were applied to each lane. The blot was hybridised with a 0.55 kb PstI-PstI *ret* cDNA probe containing part of the tyrosine kinase domain (Iwamoto *et al.*, 1990, 1991*a*). The positions of 28S and 18S ribosomal RNA are indicated.

**Figure 3** Western blot analysis of the Ret proteins. Lysates containing 20 μg of proteins from a melanocytic tumour and neuroblastoma developed in MT/*ret* transgenic mice and from normal liver and kidney of an MT/*ret* transgenic mouse were separated on 8% SDS-polyacrylamide gel and analysed by Western blotting with normal rabbit IgG (10 μg ml$^{-1}$, lanes **a**), or anti-Ret polyclonal antibody (10 μg ml$^{-1}$, lanes **b**,) 100 kDa and 96 kDa Ret proteins are indicated.

**Figure 4** Detection of phosphotyrosine-containing proteins. Lysates containing 50 μg of proteins from tissues described in Figure 3 were analysed by Western blotting with anti-phosphotyrosine antibody. A 100 kDa phosphorylated band is indicated.

detected in the lysates from normal liver and kidney of a transgenic mouse with melanocytic tumours. The same 100 kDa Ret protein was also observed in the cell lysate of the neuroblastoma, while the presence of a 96 kDa Ret protein was unclear (Figure 3). Rather, the anti-Ret antibody recognised a broad band of 93 to 100 kDa in the lysate of the neuroblastoma, suggesting that part of the Ret proteins might have been degraded.

To examine phosphotyrosine (PTYR)-containing proteins in the neuroblastoma, the lysate was reacted with an anti-PTYR antibody. As shown in Figure 4, a 100 kDa band was detected in the neuroblastoma as well as in the melanocytic tumour. On the other hand, this 100 kDa phosphorylated band was absent in the lysates from normal liver and kidney of an MT/*ret* transgenic mouse. Since the electrophoretic mobility of this band was consistent with the 100 kDa Ret protein, it is possible that the 100 kDa phosphorylated band represented the Ret protein. In addition, the level of tyrosine phosphorylation in neuroblastoma cells seemed to be lower than that in melanocytic tumour cells (Figure 4).

Although several kinds of transgenic mice carrying oncogenes driven by a metallothionein regulatory unit have been produced (Messing *et al.*, 1985; Ruther *et al.*, 1987; Heisterkamp *et al.*, 1990), there have been no reports of development of neuroblastoma. In addition, there was no spontaneous development of neuroblastoma in the MT/*ret* transgenic mice. It is known that the *ret* proto-oncogene is expressed at high levels in human neuroblastomas (Takahashi & Cooper, 1987; Ikeda *et al.*, 1990; Nagao *et al.*, 1990; Takahashi *et al.*, 1991). Thus, it is interesting that neuroblastoma developed in an MT/*ret* transgenic mouse. Since the Ret proteins are present as membrane-bound glycoproteins like the proto-Ret proteins (Taniguchi *et al.*, 1992), it is possible that both of them have similar functions in proliferation of neuroblasts. The fact that four of 17 MT/*ret* transgenic founder mice displayed disorders of melanoblasts which also originate from the neural crest cells (Iwamoto *et al.*, 1991*b*) suggested that the MT/*ret* transgene is expressed preferentially in these cell types. The sequence present in the MT/*ret* fusion gene may be responsible for this unique expression pattern in our transgenic mice. Analysis by *in situ* hybridisation is necessary to elucidate the precise expression pattern of the *ret* oncogene during embryogenesis of the MT/*ret* transgenic mice.

We thank Dr M. Hamaguchi for the anti-PTYR antibody. This work was supported in part by Grants-in Aid from the Ministry of Education, Science and Culture of Japan and the Ministry of Welfare of Japan, by the TOYOTA Foundation and by the Life Science Research Project of Institute of Physical and Chemical Research (RIKEN).

### References

CHOMCZYNSKI, P. & SACCHI, N. (1987). Single-step method of RNA isolation by acid guanidinium thiocyanate-phenol-chloroform extraction. *Anal. Biochem.*, **162,** 156–159.

HAMAGUCHI, M., GRANDORI, C. & HANAFUSA, H. (1988). Phosphorylation of cellular proteins in Rous Sarcoma virus-infected cells: Analysis by use of anti-phosphotyrosine antibodies. *Mol. Cell Biol.*, **8,** 3035–3042.

HEISTERKAMP, N., JENSTER, G., HOEVE, J., ZOVICH, D., PATTENGALE, P.K. & GROFFEN, J. (1990). Acute leukemia in *bcr/abl* transgenic mice. *Nature*, **344,** 251–253.

IKEDA, I., ISHIZAKA, Y., TAHIRA, T., SUZUKI, T., ONDA, M., SUGIMURA, T. & NAGAO, M. (1990). Specific expression of the *ret* proto-oncogene in human neuroblastoma cell lines. *Oncogene*, **5,** 1291–1296.

IWAMOTO, T., PU, M., ITO, M., TAKAHASHI, M., ISOBE, K., NAGASE, F., KAWASHIMA, K., ICHIHARA, M. & NAKASHIMA, I. (1991*a*). Preferential development of pre-B lymphomas with drastically down-regulated N-myc in the Em-ret transgenic mice. *Eur. J. Immunol.*, **21,** 1809–1814.

IWAMOTO, T., TAKAHASHI, M., ITO, M., HAMAGUCHI, M., ISOBE, K., MISAWA, N., ASAI, J., YOSHIDA, T. & NAKASHIMA, I. (1990). Oncogenicity of the *ret* transforming gene in MMTV/*ret* transgenic mice. *Oncogene*, **5,** 535–542.

IWAMOTO, T., TAKAHASHI, M., ITO, M., HAMATANI, K., OHBAYASHI, M., WAJJWALKU, W., ISOBE, K. & NAKASHIMA, I. (1991*b*). Aberrant melanogenesis and melanocytic tumour development in transgenic mice that carry a metallothionein/*ret* fusion gene. *EMBO, J.*, **10,** 3167–3175.

MESSING, A., CHEN,. H.Y., PALMITER, R.D. & BRINSTER, R.L. (1985). Peripheral neuropathies, hepatocellular carcinomas and islet cell adenomas in transgenic mice. *Nature*, **316**, 461-463.

NAGAO, M., ISHIZAKA, Y., NAKAGAWARA, A., KOHNO, K., KUWANO, M., TAHIRA, T., ITOH, F., IKEDA, I. & SUGIMURA, T. (1990). Expression of *ret* proto-oncogene in human neuroblastomas. *Jpn. J. Cancer Res.*, **81**, 309-312.

RUTHER, U., GARBER, C., KOMITOWSKI, D., MULLER, R. & WAGNER, E.F. (1987). Deregulated c-*fos* expression interferes with normal bone development in transgenic mice, *Nature*, **325**, 412-416.

SANTORO, M., ROSATI, R., GRIECO, M., BERLINGIERI, M.T., D'AMATO, G.L.-C., FRANCISCIS, V. & FUSCO, A. (1990). The *ret* proto-oncogene is consistently expressed in human pheochromocytomas and thyroid medullary carcinomas. *Oncogene*, **5**, 1595-1598.

TAHIRA, T., ISHIZAKA, Y., ITOH, F., SUGIMURA, T. & NAGAO, M. (1990). Characterization of *ret* proto-oncogene mRNAs encoding two isoforms of the protein product in a human neuroblastoma cell line. *Oncogene*, **5**, 97-102.

TAKAHASHI, M., BUMA, Y. & HIAI, H. (1989). Isolation of *ret* proto-oncogene cDNA with an amino-terminal signal sequence. *Oncogene*, **4**, 805-805.

TAKAHASHI, M., BUMA, Y., IWAMOTO, T., INAGUMA, Y., IKEDA, H. & HIAI, H. (1988*a*). Cloning and expression of the *ret* proto-oncogene encoding a tyrosine kinase with two potential transmembrane domains. *Oncogene*, **3**, 571-578.

TAKAHASHI, M., BUMA, Y. & TANIGUCHI, M. (1991). Identification of the *ret* proto-oncogene products in neuroblastoma and leukemia cells. *Oncogene*, **6**, 297-301.

TAKAHASHI, M. & COOPER, G.M. (1987). *ret* transforming gene encodes a fusion protein homologous to tyrosine kinases. *Mol. Cell Biol.*, **7**, 1378-1385.

TAKAHASHI, M., INAGUMA, Y., HIAI, H. & HIROSE, F. (1988*b*). Developmentally regulated expression of a human 'finger'-containing gene encoded by the 5' half of the *ret* transforming gene. *Mol. Cell Biol.*, **8**, 1853-1856.

TAKAHASHI, M., RITZ, J. & COOPER, G.M. (1985). Activation of a novel human transforming gene, *ret*, by DNA rearrangement. *Cell*, **42**, 581-588.

TANIGUCHI, M., IWAMOTO, T., NAKASHIMA, I., NAKAYAMA, A., OHBAYASHI, M., MATSUYAMA, M. & TAKAHASHI, M. (1992). Establishment and characterization of a malignant melanocytic tumor cell line expressing the *ret* oncogene. *Oncogene*, **7**, 1491-1496.

WAJJWALKU, W., TAKAHASHI, M., MIYAISHI, O., LU, J., SAKATA, K., YOKOI, T., SAGA, S., IMAI, M., MATSUYAMA, M. & HOSHINO, M. (1991). Tissue distribution of mouse mammary tumor virus (MMTV) antigens and new endogenous MMTV loci in Japanese laboratory mouse strains. *Jpn. J. Cancer Res.*, **82**, 1413-1420.